# An Overview of the Microbiota of the Human Urinary Tract in Health and Disease: Current Issues and Perspectives

**DOI:** 10.3390/life13071486

**Published:** 2023-06-30

**Authors:** Marica Colella, Skender Topi, Raffaele Palmirotta, Donato D’Agostino, Ioannis Alexandros Charitos, Roberto Lovero, Luigi Santacroce

**Affiliations:** 1 Microbiology and Virology Unit, Interdisciplinary Department of Medicine, University of Bari “Aldo Moro”, Piazza G. Cesare, 11, 70124 Bari, Italyraffaele.palmirotta@uniba.it (R.P.); 2 Department of Clinical Disciplines, School of Technical Medical Sciences, University of Elbasan “A. Xhuvani”, 3001 Elbasan, Albania; skender.topi@uniel.edu.al (S.T.);; 3 Respiratory Rehabilitation Unit, Clinical Scientific Institutes Maugeri (IRCCS), Section of Bari, 70124 Bari, Italy; 4 AOU Policlinico Consorziale di Bari-Ospedale Giovanni XXIII, Clinical Pathology Unit, Policlinico University Hospital of Bari, 70124 Bari, Italy

**Keywords:** human microbiota, urinary system microbiota, immune modulation, dysbiosis, probiotics, urinary system diseases, urgency incontinence, urinary tract infections, bladder cancer, estrogen metabolism

## Abstract

This article is intended to deepen our knowledge to date regarding the functions of the resident microbiota/microbiome in the urinary system for human health and disease. First, we sought to report the general characteristics (composition and stability) of the normal urinary system microbiota in the different anatomical sites in relation to some factors such as the effect of age, gender and diet, analyzing in detail the functions and the composition of the microbiota in the light of current knowledge. Several pieces of evidence suggest the importance of preserving the micro-ecosystem of the urinary system, and in some cases their relationship with diseases is important for maintaining human health is well understood. The female and male reproductive microbiota have mainly been studied over the past decade. In the past, the arrest was thought to have taken place in a sterile environment. Microorganisms of the microbiota form biofilms, three-dimensional structures, that differ in the reproductive organs and interact with both gametes and the embryo as well as with maternal tissues. These biofilms from the reproductive system also interact with others, such as that of the gastrointestinal tract. Reduction in its diversity intestinal microbiota can disrupt estrogen metabolism and affect the reproductive microbiota. It is therefore understood that its quantitative and qualitative identification is important for microbiota, but also the study of the structures formed by the microorganisms. A dysbiosis with local or systemic causes can lead to serious diseases. The role of probiotics in maintaining microbial population harmony (eubiosis) and preventing certain pathologies of the urinary and reproductive system was also investigated. A negative variation in the qualitative and quantitative composition of certain strains of microorganisms (dysbiosis) due to local or systemic causes can even lead to serious diseases. The role of probiotics in maintaining the healthy balance of microorganism populations (eubiosis), and thus in the prevention of certain pathologies of the urinary and reproductive system, has also been studied.

## 1. Introduction

It is exciting to see how our perceptions of microorganisms have evolved from Pasteur and Koch’s original claims that showed “germs” as the basis of disease in the late 18th century, but in the present era there is a multitude of microbes, which constitute a natural microbial population. Later, such special physiological bacterial populations, that we call resident human microbiota and which are part of our organism, provide fundamental health balance support for our well-being. Until recently, urine was considered sterile to germs in healthy individuals. Their composition does not favor the growth of microorganisms because they have increased salinity, relatively low pH (average 6.0), high concentrations of urea, etc. Even the nitrates in the slightly acidic environment inhibit the growth of some uro-pathogenic bacteria, but there are some proteins that also have antimicrobial activity. Moreover, the continued removal of urine from the organism does not favor the growth of microorganisms. All microorganisms that survive under these conditions must have developed special adaptation mechanisms, e.g., S. saprophyticus tolerates high concentrations of D-serin, which is abundant in the urine with antimicrobial activity against organisms lacking D-serine deaminase. The bladder was not initially examined because it was considered sterile and there were also difficulties in collecting samples. It has now been found that healthy people have a resident microbiota of normal flora in their urinary tract, dispelling the old belief that finding microorganisms in urine meant an infection.

Their protective role in maintaining health and their involvement in ailments and diseases is slowly becoming clearer [1,2,3,4].

*Current microbiology diagnostic tests*

The microscopic examination provides useful information mainly for a presumptive diagnosis, which must then be subsequently confirmed. An overview of the main microbiology tests currently used for diagnosis of infectious conditions is summarized in Table 1:

- The sample can be observed: (a) after staining, for the targeted search for specific microbial/pathogen groups (i.e., Gram staining)., (b) “fresh observation” without staining. They are both suitable for the study of some biological properties (shape, organization, motility, chemical reactivity);
- The choice of the microscopic technique to be used depends on the pathogen whose presence is suspected: (a) bright field microscopy (frequent use), (b) dark field microscopy (e.g., for *Treponema pallidum*), (c) phase contrast microscopy (frequent use), and (d) fluorescence microscopy (greater sensitivity because antibodies are used to search the microorganism)
- With advanced cultivation techniques and molecular approaches, wide numbers and variations have been identified types of microorganisms in healthy and asymptomatic individuals.

Therefore, one of the main reasons why urine from healthy patients has traditionally been considered sterile was the study of urine using standard cultures techniques. These methods only allow the growth of a very limited number of bacteria, including *Escherichia coli*, the main UTI-causing uropathogen. The microbiota of the reproductive system, especially the vaginal one, helps the body defend itself and maintain its pH. Furthermore, one of the main functions of the microbiota of the reproductive system is transmembrane transport and the metabolism of amino acids and carbohydrates [5]. Therefore, a separate and combined study of the ovarian microbiota with the ovaries and oocytes, the testes with the sperm, and finally their course and/or the newly formed embryo in the fallopian tubes, uterus, cervix and vagina was conducted [6]. In the reproductive system, the vagina was mainly studied, where bacteria of *Bacillota* phylum predominate. Thus, each stage of human reproduction is performed in a different compartment and is accompanied by a variety of microorganisms. Samples from cohorts of pregnant women show an increase in the relative abundance of *Bacillota* phylum in vaginal microbiota from the first to the third trimester of pregnancy [5,6].

## 2. The Microbiota Composition of the Urinary Tract

The kidneys, ureters, urinary bladder and urethra make up the urinary tract. Conventional urine culture methods were designed mainly for the growth of aerobic microbes and pathogens, e.g., *Escherichia coli*, and, on the contrary, recently, the application of molecular techniques has provided a more complete picture of the microbial community in the urinary system. In the urethra, the microbiota is mainly constituted of microorganisms of the *Lactobacillaceae* family, coagulase-negative *Staphylococcus* (CoNS), *Streptococcus* (viridans, non-hemolytic) spp., *Lactobacillus* spp., *Diphtheroids*, non-pathogenic *Neisseria*, *Coccobacilli*, *Mycoplasma* spp., some anaerobic bacteria and, occasionally, *Saccharomyces* [1,2]. As the symbiotic microorganisms of the bladder include bacteria such as those from the *Lactobacillaceae* family *Lactobacillus*, *Staphylococcus* and *Gardnerella* which play a protective role in the development of pathogens, it is therefore maintaining homeostasis in the urinary system (Table 2). However, there is continuous new research on the variability of the urinary tract microbiota communities and their effect on health, then between eubiosis and dysbiosis. Scientific research on the urinary tract microbiota currently aims to understand the role that can play in the development of urinary tract infections (UTI) but also in other diseases such as in UI, cancer and others [4,7,8].

The urinary microbiota differs between men and women, possibly due to anatomical differences. However, this can also be due to different molecules in the urine of the two genders. Women produce more citrate but less calcium and oxalate, while men secrete more creatinine. The different components promote the growth of specific bacteria (Table 3).

**Figure 1:**
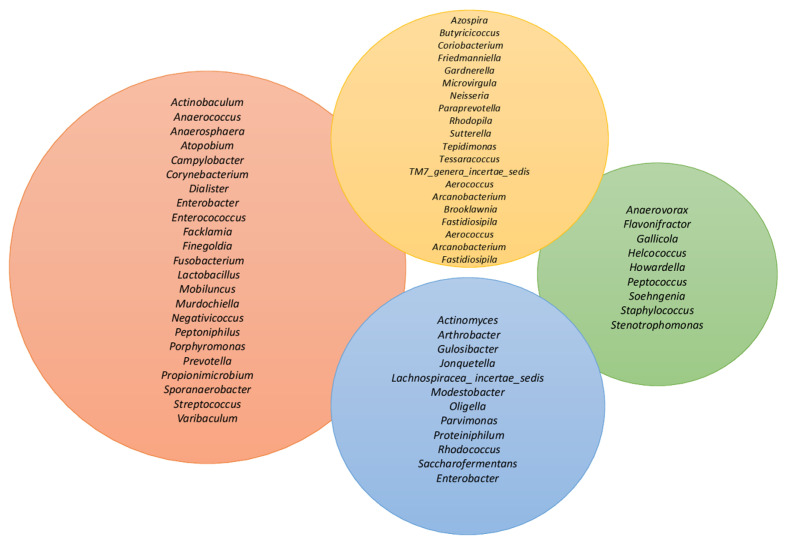
There are differences in composition between the microbiota and age. The figure indicates the microbial composition between two age groups (20–49 and age ≥70) on a certain sample of women without urinary pathologies. The orange circle indicates the common bacteria found in all ages groups while the green circle indicates the common bacteria, between the two age groups (20–49 and age ≥70). The yellow circle indicates bacteria present in the age range 20–49 and the blue circle the age range ≥70 [16].

Other molecularly identified microbes (16S rRNA gene sequencing in voided urine of asymptomatic adults) in female samples included representative members of *Actinobacteria* (e.g., *Actinomyces*, *Arthobacter*) and *Bacteroides* sometime absent from male samples [2,9,10,11,12]. There are fewer reports on the urinary microbiota in men. In a study that used a 16S rRNA gene sequence to characterize bacterial communities in the coronal sulcus and distal urethra in urine collected from adolescent boys, *Streptococcus*, *Gardnerella*, *Lactobacillus* and *Veillonella* were dominant. *Streptococcus*, and to a lesser extent *Veillonella*, have repeatedly been observed in urine of healthy men [11,13]. Regarding the differences in the urinary microbiota between the genders, some researchers who used the 16S rRNA sequence from urine samples concluded that the microbiota is characterized by the predominance of *Lactobacillales* in women and *Corynebacterium* in men [13]. The microbiota in the urine does not remain constant but changes with age. Children and adults have different populations of microorganism germs, which can be due to the influence of hormones, change in diet, personal hygiene, etc. There are different bacterial genera in age groups among adults. Using molecular analyses, one study found four genera that were present exclusively in the elderly (>70 years) regardless of gender: *Jonquetella*, *Parvimonas*, *Proteiniphilum* and *Saccharofermentans* (Figure 1). These bacteria cannot be cultivated because they are anaerobic and have special nutritional needs. The effects of these microorganisms on the urinary tract of the elderly are still unknown and further studies are needed [6,13,14,15].

## 3. The “Behavior” of the Microbiota in Urinary Tract Diseases

Several studies have observed changes in the urinary microbiota among patients with urological disorders of the upper (such as chronic renal insufficiency) and lower urinary tract in relation to the healthy population. Lower urinary tract symptoms (LUTS) indicate a set of symptoms of the lower urinary tract, in both men and women. They can be attributable to numerous causes such as aging, diabetes, benign prostatic hyperplasia, vesico-urethral pathology (urolithiasis, bladder neoplasms, urethra-prostatitis and urinary infections, urethral stenosis, hyperactivity/hypoactivity of the bladder detrusor muscle, neurogenic bladder dysfunction and other [16,17].

### 3.1. Role of the Microbiota Gut/Kidney and Bladder/Gut/Brain Axes

The microbiota is a complex interconnected bio-system of microorganisms in the human body. The most important is the intestinal one which maintains its relationship with various organs of the body thus maintaining a dynamic equilibrium (eubiosis) [1]. The urinary tract has its own specific microbiota. In fact, it has higher levels of *Actinomycetota*, *Fusobacteria* and *Pseudomonadota* but lower levels of *Bacteroidota*, *Bacillota* and *Verrucomicrobiota* phyla compared with the intestinal microbiota [1,2]. Thus, intestinal microbiota contains a huge number of bacteria that coexist with humans playing a key role in homeostasis. Intestinal colonization begins immediately after birth and is influenced by many factors, such as diet (breastfeeding, formula milk), geographical location, age and the use of antibiotics [18,19]. In fact, the gut connects the interaction between the microbial components, and which interacts with the different organs of the host. We can therefore distinguish the cross-talking microbiota axes between gut/lung, gut/brain, gut/skin, bladder/gut/brain and gut/kidney axes. This has the effect of not causing diseases on the microbiota but allows the microbiota itself to prevent the growth of pathogenic microorganisms helping the host to have a ready and effective strong immunity [19,20,21,22,23]. This knowledge contributes to the correct causal-rational use of antimicrobial drugs and to the interpretation of cultures of various biological samples. The gut–kidney axis could be implicated in acute kidney injury, chronic kidney disease, urolithiasis, IgA nephropathy, and others. It has been noted that during an involving kidney disease (such as an end-stage chronic kidney disease and urolithiasis) the microbiota is overpopulated by *Enterobacteriaceae* and *Streptococcaceae*, with less presence of *Prevotellaceae* and *Roseburia*. During chronic renal failure, the intestinal microbiota through its urease transforms urea into ammonia. Then begins a biochemical process which will result in the production of uremic compounds and toxins (such as noxyl sulfate, p-cresyl sulfate (PCS) and trimethylamine (TMA) N-oxide). In turn, this condition and an increase in the presence of urea will lead to a worsening of the dysbiosis of the intestinal friendly bacterial populations such as *Lactobacillus* spp. and *Bifidobacterium* spp. from the *Lactobacillaceae* family. Thus, the balance of all the axes with the gut is altered [19,24].

The hypothesis of a bladder–intestine–brain axis is useful because it is possible to investigate the pathophysiology of functional, related urological and gastrointestinal disorders because they could play a role in the rise in the formation of clinical signs and symptoms [25,26,27]. It is hypothesized that a dysregulation of this communication pathway can result in a state of perceived hypersensitivity to the visceral stimulus which results in an amplified sensitivity, such as hyperalgesia in slightly painful response, or of allodynia in response to painless stimuli. Furthermore, this dysregulation can cause an unjustifiable emotional/cognitive imbalance (anxiety, asthenia, negative mood, hypervigilance, pain and more) which is in the cortico–amygdala pathway and in the reward circuits of the cortico–basal ganglia [24,27]. This condition could contribute as a negative factor leading both to emotional distress with psychiatric aspects and disorders but also to bodily discomfort with the manifestation of functional disorders. This hypothesis could include the role of the microbiota. If there is a possible variation with loss of diversity of microorganisms (dysbiosis), such as acute or chronic inflammatory conditions in the intestine, commensal bacterial strains act in favor of the inflamed mucosa. This occurs because they inhibit the IL-6 and NFκB signaling pathways [27]. Furthermore, there is also a gut/brain crosstalking. This axis includes the neuro-intestinal system and the hypothalamic–pituitary–adrenal axis, the central and peripheral nervous system and the autonomic sympathetic system. Immune function, mucus production, microbiota composition and intestinal motility and permeability vary under conditions of a stress-inducing stimulus. The intestinal microbiota, in turn via the intestinal nervous system and sympathetic/parasympathetic pathways, activates: (a) the production of neurotransmitters (GABA, serotonin, etc.) and neurotrophic factor (BDNF), (b) the modulation of enteric sensory afferences, (c) the production of the bacterial metabolites, (d) the intestinal barrier protection, (e) the tight junction’s integrity and (f) the immune regulation [28]. Thus, changes in the composition of the urinary microbiota also related to inflammation and, since affective disorders occur via inflammatory pathways, can cause affective and/or functional disorders that are both immunologically related to dysbiosis [13,19].

### 3.2. Urinary Tract Infections (UTIs)

Urinary tract infections (UTIs) can affect any organ of the urinary system (kidneys, ureters, bladder and urethra). However, most infections involve the lower urinary tract (bladder and urethra). The most vulnerable groups are women, due to their anatomy, and the elderly. The main pathogen for UTIs was considered *E. coli*, but recent data have identified other organisms associated with these conditions, such as *Streptococcus* spp. and *Enterococcus* spp. In addition, other causative agents were *Enterococcus faecalis*, *Klebsiella pneumoniae* and *Proteus mirabilis* (Table 4) [1,11,16,18,29].

The *Escherichia coli* infections account for 70–95%, *Staphylococcus* spp. 5–20%, Proteus mirabilis and *Klebsiella*, *Enterococcus* spp. 1–2% of UTIs. Instead, *Citrobacter* spp., *Enterobacter* spp., *Pseudomonas aeruginosa*, group B *Streptococcus*, *Staphylococcus aureus* and other represent <1% of urinary infections. Finally, *Escherichia-Shigella* taxa, which are potentially responsible for urinary tract infections, are present in the urinary microbiota in elderly women. Molecular techniques enable the detection of a wider range of bacterial genomic sequences, some of which identify species that cannot be cultivated (Table 5) [4,10,12,29,30,31].

In a study of eight female patients with interstitial cystitis (IC) the microbiota was composed by *Lactobacillaceae* family, *Lactobacillus*, *Gardnerella*, *Corynebacterium*, *Prevotella*, *Ureaplasma*, *Enterococcus*, *Atopobium*, *Proteus* and *Cronobacter* through clean-catch midstream urine samples [10]. There is evidence that there may be a reservoir of pathogens in the urinary tract, resulting in recurrent infections. While other germs that live in the urogenital system are destroyed by antibiotics, pathogens can penetrate deeper to stay there and cause infection in their host again. As the population of *E. coli* resulting in reinfection, the percentage of *Lactobacillus* species from *Lactobacillaceae* family normally present in the urogenital system is significantly reduced. Indeed, two *Lactobacillus* species predominate in healthy females: *Lactobacillus crispatus* and *Lactobacillus iners*. These species play an important role in maintaining the health of the urinary system by producing metabolic products (such as lactic acid, hydrogen peroxide) that interfere with the adhesion of *E. coli* and help to reduce its infectivity [32,33].

As mentioned, microbiota from both small and large intestine have several bacterial populations and, thanks to the presence of the intestinal mucosal barrier, prevents them from the translocation to other organs. However, there may be certain favorable conditions that allow the spread of certain bacterial strains can take place under certain favorable conditions. Microorganism bowel movement (MBM) or bacterial translocation is defined as the phenomenon in which live bacteria, their derivatives or both pass through the intestinal mucosal barrier (IMB) and colonize extra-intestinal tissues such as those of the urinary tract [34,35].

The mesentery lymph nodes are the first extra-intestinal organ to colonize, followed by the liver, spleen and systemic circulation. There are three main mechanisms that promote MBM: (a) overgrowth of the intestinal microbiota such as the small intestinal bacterial overgrowth (SIBO), (b) immune deficiency states, and (c) damage to the intestinal mucosa architecture. When one of these mechanisms is of particular impact, its action is prolonged or combined with another, increases the severity of MBM and can lead to severe sepsis, and also multi-organ failure (MOF) [36]. The normal intestinal microbiota and the intestinal immune system are interdependent. Through the local production of anti-inflammatory molecules and the induction of apoptosis, the intestinal wall copes with constant antigenic irritation [37]. When the intestinal mucosa is stimulated by intestinal lumen antigens, the local products of immune system cells (T lymphocytes, B lymphocytes, monocytes/macrophages and dendritic cells) begin to enter the lymphatic circulation and through the thoracic duct reach the systemic circulation via the which return to the mucosal epithelium. T lymphocytes produce cytokines (IL-4, IL-5, IL-6, IL-10) which in turn stimulate the production of IgAs by mature B lymphocytes. Auxiliary T cells via IFN-γ and lymphotoxins (LTs), such as α (LTα) and β (LTβ), in turn balance this demand. T lymphocytes account for 1/3 of epithelial cells with a CD4(+)/CD8(+) ratio such as that of peripheral blood. T lymphocytes CD4(+) of the epithelium are memory cells and produce large amounts of cytokines (IFN-γ, IL-12, IL-18) [38,39,40,41,42].

Experimental studies have shown that the most common microorganisms involved in MBM are *Candida* spp., *Escherichia coli*, *Klebsiella pneumoniae* and *Proteus mirabilis*. Although anaerobic bacteria make up most of the endothelial microbiota, they have not been described as associated with MBM in the blood, possibly due to the presence of oxygen in the blood which inhibits their growth. Finally, there is an inverse relationship between the presence of *Oxalobacter formigenes* in the intestinal microbiota and the formation of calcium oxalate kidney stones. *Oxalobacter formigenes* is essential for the breakdown of dietary oxalates in the intestine. Colonization with the bacterium reduces the risk of recurrence of calcium oxalate stones by 70%. Patients with urolithiasis have been noted to have half the population of the bacterium in respect to healthy individuals [43,44].

Finally, sexually transmitted infections (STIs) can be caused from protozoan parasites (*Trichomonas vaginalis*), bacteria (*Chlamydia trachomatis*, *Neisseria gonorrhoeae*, *Treponema pallidum* subsp. *pallidum*, etc.), viruses such as Herpes simplex II, hepatitis B, human papillomavirus, HIV and fungi such as *Candida albicans*. They may does not cause symptoms immediately and sometimes share many similar symptoms with urinary tract infections, including a burning sensation and/or pain in the lower abdomen, fever. In some comparative studies in male individuals without and with STIs, the microbiota was studied, noting the presence of *Lactobacillus*, *Sneathia*, *Gemella*, *Aerococcus*, *Corynebacterium*, *Streptococcus*, *Veillonella*, *Prevotella*, *Anaerococcus*, *Propionibacterium*, *Atopobium*, *Staphylocoplasma*, *Aerococcus*, *Enterococcus*, *Finegoldia*, *Neisseria*, *Propionibacterium* and *Ralstonia* in the first-void urine sample collection [45,46].

### 3.3. Urgency Urinary Incontinence (UUI)

Urinary incontinence (UI) and urgency urinary incontinence (UUI) or overactive bladder (OAB) are very common, but not clearly understood, conditions characterized by urgency, frequency and urinary incontinence that mainly affects women and the elderly. The hypotheses to explain the etiology of UI and OAB are: (a) reduction in sensory triggers that decrease inhibitory tone of the detrusor muscle, (b) possible peripheral or central nervous system deficit that causes a reduction in inhibitory control of the detrusor, (c) hyperactivity of the pelvic floor due to increase in afferently inputs from urethra, bladder and pelvic floor. Risk factors may be obesity, menopause, poor pelvic floor tone, multiple natural childbirths, excessive consumption of stimulants (such as caffein, taurine and other), cigarette smoke and urological interventions [47]. Therefore, the incidence of UIs in women increases after menopause, so age and estrogen are thought to play a role. The urinary microbiota microbial flora is likely to change as it changes with age and is also affected by estrogen. In fact, other microbial areas, such as the vaginal tract, are significantly compromised in the absence of estrogen. Since estrogen not only alters the bladder epithelium but also increases the likelihood of UTI and UUI incidence, the microbiota is likely to change after menopause and can cause many symptoms associated with menopause. As the urinary microbiota appears to play a protective role, changes in its microbial composition are an additional factor some people have experienced urgent urinary incontinence [1,48].

Since there is significant overlap between symptoms of UUI and those of UTIs, it is possible that UUI has a microbial etiology. Urinalysis with urine culture may detect the presence of a urinary tract infection (which generally has the same symptoms as painful overactive bladder) and which, in turn, may be the cause of secondary overactive bladder syndrome. One study compared the urinary microbiota of women with UUI to that of women without UUI and statistically significant differences in the frequency and abundance of existing bacteria. These differences suggest a possible role of the urinary microbiota in maintaining urinary tract health in women. Some urine samples were dominated by the *Lactobacillus* or *Gardnerella* genera. or the *Enterobacteriaceae* family, which includes *Escherichia*, *Klebsiella* and *Proteus*. Instead, in a study, recruiting eleven female patients with UI and twelve with pelvic organ prolapse (POP), it was noted that the microbiota was composed by species from *Lactobacillaceae* family *Lactobacillus*, *Actinobaculum*, *Aerococcus*, *Anaerococcus*, *Atopobium*, *Burkholderia*, *Corynebacterium*, *Gardnerella*, *Prevotella*, *Ralstonia*, *Sneathia*, *Staphylococcus*, *Streptococcus* and *Veillonella* with the by clean-catch midstream urine, suprapubic aspirate and transurethral catheter sampling methods [2,10]. In other samples, no bacteria predominated. These have been called “miscellaneous” samples. Although *Lactobacillus* was isolated from both groups, there were species-level differences, with *Lactobacillus gasseri* being most often detected in the UUI group and *Lactobacillus crispatus* being most frequently detected in controls. Hence, these data suggest that there are potentially significant differences in the urinary microbiota of women without and with UUI (dysbiosis is identified). This condition could add value in the prevention, diagnosis and treatment of UUI. Indeed, therapy for UUI which consists of behavioral therapy (special diet avoiding irritants and other), perineal physio-kinesitherapy and pharmacological therapy (using antimuscarinics that act by blocking the involuntary contractility of the detrusor), and the adjuvant role of probiotics/prebiotics would be important [48,49,50,51,52].

### 3.4. Bladder Cancer

Bladder cancer has been linked to a variety of risk factors, including hormones, tabagism and several infections such as *Schistosoma haematobium*, and has mainly been observed in people with asymptomatic bacteriuria. Thus, the microbiotal variations in patients with urothelial carcinoma compared to that of healthy individuals is an additional risk factor. There are many carcinogenic substances that circulate in the intestine and pass into the blood which is filtered by the kidneys and is stored in the bladder before excretion, and these can affect the urinary microbiota. It is highly likely that some members of the commensal microbiota can isolate toxic substances, such as heavy metals and aniline, that are considered risk factors for bladder cancer [53,54].

**Figure 2:**
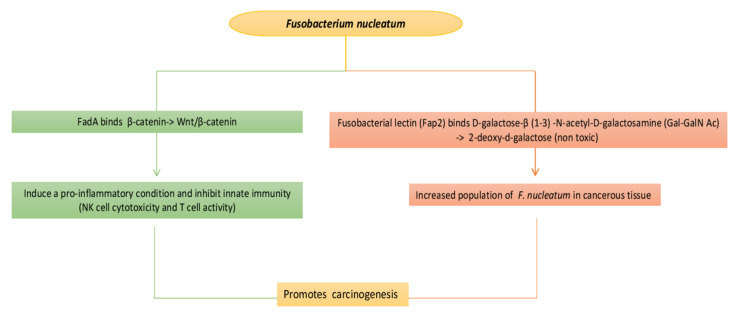
One possible mechanism or the carcinogenesis from *F. nucleatum*. FadA, *F. nucleatum* adhesin, binds to β-catenin on the surface of tumor cells and activates oncogenic Wnt/β-catenin signaling. *F. nucleatum* can also affect the function of lymphocytes and natural killer cells by binding to the TIGIT receptor (expressed on all NK cells, as well as on other immune cells) and through another adhesion FAP2 also binds to a disaccharide (Gal-GalNAc), which is expressed on the surface of several neoplastic and other cells, facilitating the binding of *F. nucleatum* to colon cancer cells.

Urinary carcinoma is the main type of cancer located in the urinary system, with the most common area of manifestation being the bladder. Changes in the urinary microbiota are highly likely to represent a new risk factor for bladder cancer, in proportion to the involvement of the microbiota in developing tumors elsewhere in the body. A small sample study showed enrichment with *Acinetobacter* which was the predominant genus and *Streptococcus* in the urine of urothelial carcinoma patients. Other bacteria that they linked with precancerous and cancerous conditions are the increase in *Fusobacterium* (common in oral microbiota, sometimes opportunistic pathogen and associated with esophageal mucosal alterations and colorectal cancer), *Campylobacter hominis*, *Jonquetella*, *Anaerococcus*, *Sphingobacteriumm*, *Herbaspirillum*, *Porphyrobacter* and *Bacteroides* have been associated with the tumor [55,56]. Indeed, a microbiota analysis study suggests that bladder cancer tissue may also be colonized by *Fusobacterium nucleatum*. Carcinogenesis promoted by *F. nucleatum* probably occurs via the cellular signaling pathway of β-catenin and activates oncogenic Wnt/β-catenin signaling which will lead to cell multiplication generating a proinflammatory condition. This can affect the function of lymphocytes and natural killer cells by binding to the TIGIT receptor (expressed on all NK cells, as well as on other immune cells) and inhibit innate immunity (NK cell cytotoxicity, T cell activity, etc.), there by promoting immune evasion, which is one of the hallmarks of cancer that helps the tumor to proliferate. The increased population of *F. nucleatum* in cancerous tissue appears to be manifested by fusobacterial lectin (Fap2) which binds with D-galactose-β (1–3) -N-acetyl-D-galactosamine (Gal-GalN Ac) and which is present in various tumor type, but only moderate levels have been found in urothelial carcinomas (Figure 2) [1,36,54,55,56,57].

*Campylobacter* is potentially pathogenic, as some studies have shown, and the species are able to produce toxins and invade epithelial cells, eluding the immune response. The presence of *Jonquetella* is potentially characteristic of the urinary microbiota of individuals aged 70+ years and over, and this supports the theory that aging changes the composition of microorganism communities both in the bladder and whether this variation increases the risk for cancer remains to be investigated [57,58].

Finally, the importance of manipulation of the bladder microbiota is considered during intravesical treatment of urothelial cell carcinoma (UCC) by instillation immunotherapy with the bacillus of Calmette-Guerin (BCG, attenuated form of the bacterium *Mycobacterium bovis*). BCG adheres to transitional cells through a fibronectin-dependent mechanism (the FAP receptor present on the *Mycobacterium* is responsible for adhesion to fibronectin), the mycobacterium-receptor complex is phagocytized inside the cells where it is processed and subsequently presented to the external surface for immune recognition. Subsequently, a wide number of immune system cells are activated (macrophages, T and B lymphocytes, natural killer (NK), BCG-activated killer cells); in the infiltrate at the level of the bladder wall there is a predominance of T lymphocytes, and there is a significant increase in cytokines. The response of T helper 1 (Th-1) lymphocytes is associated with the effective elimination of tumor cells. The cytokines produced are represented by interferon-gamma (IFN-γ), tumor necrosis factor-alpha (TNF-α) and interleukins (IL-1, IL-2, IL-6, IL-8, IL-12) which amplify the number of cytotoxic cells capable of attacking dump kinetic cells [39]. IFN-γ is an immunostimulatory lymphokine that increases the immune class I and II major histocompatibility complex (MHC), the expression of Fc receptors, and expresses an antiangiogenic effect. The IP-10 is the only chemokine that attracts activated T lymphocytes and is a potent inhibitor of angiogenesis, tumor growth and metastasis in vivo [51,54,55,56,57,58].

## 4. The Microbiota of Female and Male Reproductive System

The microbiota of reproductive system can be studied separately but also combined, e.g., the ovary with the ovaries and oocytes, the testes with the sperm and their ducts and/or the embryo in the fallopian tubes, in the uterus, in the cervix and vagina. The microbial layers of the biofilms differ in the reproductive organs and interact with both gametes and the fetus, as well as with maternal tissues [5,59,60]. Moreover, these biofilm layers interact with others, such as that of the gastrointestinal tract. Reduction in diversity in intestinal microbiota can disrupt estrogen metabolism and affect the reproductive microbiota [5,59,61].

Vaginal microbiota is characterized by the lowest α and β diversity and it is dominated by *Lactobacillus* spp. (106–108 cfu/g) that act as probiotics and limit the spread of other bacteria [62]. Therefore, *Lactobacillus* spp. implement several mechanisms to carry out a protective effect in defense of the vaginal mucosa from the aggression of pathogenic microbes by the inhibition of the growth, adhesion and multiplication. The vaginal microbiota in fertile age (the so called flora of Doderleïn) is dominated by the presence of different *Lactobacillus* species from the *Lactobacillaceae* family such as *Lactobacillus acidophilus*, *Limosilactobacillus fermentum* (aka *Lactobacillus fermentum*), *Lactiplantibacillus plantarum* subsp. *plantarum* (aka *Lactobacillus plantarum*), *Levilactobacillus brevis* (aka *Lactobacillus brevis*), *Lactobacillus jensenii*, *Lacticaseibacillus casei* (aka *Lactobacillus casei*), *Ligilactobacillus salivarius* (aka *Lactobacillus salivarius*) [63,64,65,66]. It distinguishes five types of vaginal colonization according to the English Community State Type (CTS) (Table 6). The group IV from some studios is divided into two or three groups that contain mandatory and optional anaerobic bacteria from *Clostridiales*, *Gardnerella* and a few *Lactobacillus* spp.

**Figure 3:**
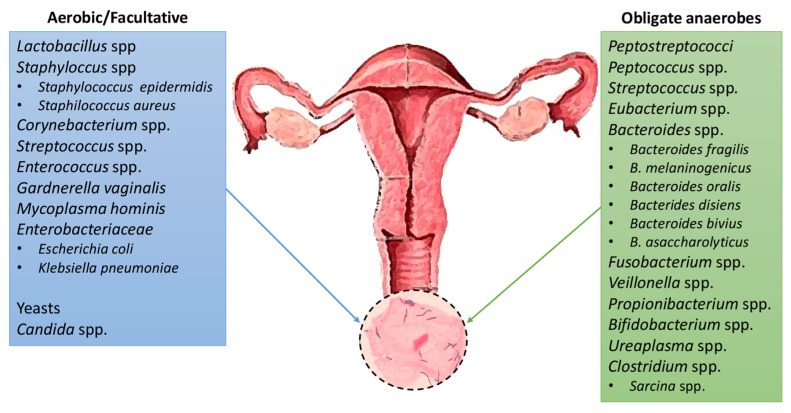
Microbial species detected among definitive studies in the microbiota of the nomal human vagina.

Nevertheless, its composition may vary between menstrual periods, sexual intercourse and menopause, or it may differ between races (Figure 3) [62,67,68,69].

Inhibition of the growth of pathogens takes place via the *Lactobacillus* genus that produce lactic acid (pH 3.5–4.0), H_2_O_2_ (with bactericidal effect on some strains), bacteriocins (antibiotic action) and by the nutritional competition sources (e.g., arginine deprivation for anaerobes) [62,63,70]. The biofilm of the vagina extends to the endometrium, but also in the fallopian tubes. This ensures a healthy environment for achieving pregnancy implantation and further embryonic development. Postgenomic studies indicate that the vaginal microbiota between healthy and asymptomatic women may differ [69,71,72]. The healthy microbiota includes one or two predominant *Lactobacillus* species from the *Lactobacillaceae* family, mainly *Lactobacillus iners* or *Lactobacillus crispatus*. Other often dominant species are *L. gasseri* and *L. jensenii* (from samples of women undergoing in vitro fertilization, or IVF) [62,73].

Asymptomatic women also show bacteria of the genus *Atopobium*, *Gardnerella*, *Prevotella* and *Megasphaera*. The dominance of *L. crispatus* has been shown to contribute to the most effective maintenance of a healthy vaginal microbiota eubiosis. The presence of *Ureaplasma* in the vagina appears to be more common in infertile women [74,75,76]. The presence of *Staphylococcus* spp. and *Enterobacteriaceae* in the embryo transfer catheter and generally high microbial diversity has been associated with low implantation rates in women undergoing IVF [77,78]. Most assisted reproduction techniques involve ovary stimulation. Indeed, variations in estrogen levels appears to affect the vaginal microbiota, reducing *Lactobacillus* species from *Lactobacillaceae* family, thus increasing the susceptibility to bacterial vaginosis, and reducing its probability of pregnancy [78,79]. This condition in the vaginal microbiota may can lead to endometritis, peritoneal inflammation and premature birth, that affect the entire reproductive system [80,81]. Moreover, women under IVF with bacterial vaginosis show reduced conception rates and an increased chance of miscarriage showed that species from *Lactobacillaceae* family had a different distribution among women with idiopathic or infertility. *L. iners*, *L. crispatus* and *L. gasseri* were sufficient to separate women with idiopathic infertility from the other. Finally, the urinary virome is represented by viruses mainly belonging to the *Papillomaviridae* family, such as HPV-16, HPV-18 and HPV-45. Women infected with human immunodeficiency virus (HIV) are at high risk of HPV infection and cervical cancer [5,82,83].

Initially the identification of endometrial microorganisms was attempted with standard microbial culture techniques of samples taken during the hysterectomy procedure with doubt in their possible contamination by the vaginal microbiota [84,85,86]. The vaginal fluid was examined by pyrosequencing of the 16S rDNA. The pyrosequencing for 16S rRNA regions identified two main types of microbiota, depending from the dominant populations of *Lactobacillus* species from *Lactobacillaceae* family. [87,88]. Like vaginal microbiota, the absence of *Lactobacillus* spp. in a receptive endometrium of women undergoing IVF was independent of hormonal fluctuations and corresponded to statistically significantly reduced rates of implantation [88]. Endometrial microbiota seems to have an immune and metabolic effect on the fetal microbiota (according to some studies that support its presence) during pregnancy [89]. The colonization of the upper genital tract by microorganisms was a pathological finding. A study has tested the vaginal microbiota and endometrium for hysterectomy for 12 bacterial species. The PCR procedure identified that most of the women (over 90%) had at least one bacterial species from the upper genitalia, with *Lactobacillus* spp. and *Prevotella* spp. being the most common. It was observed that the vaginal microbial community was significantly larger [5,87,88]. The endometrial microbiota appears to influence immunologically and metabolically the fetal microbiota during pregnancy [89,90]. Thus, the childbirth method subsequently appears to influence the neonatal microbiota [91,92]. Microorganisms have been found in the majority of placenta, amniotic fluid and umbilical cord, indicating the potential for innate fetal immunity and bacterial tolerance from the newborn [59,93]. Exploration of the endometrial microbiota in infertile women has revealed adverse effects on the functioning of the reproductive system. Successful embryo transfer depends on many factors including the presence of microbiota in the upper genitalia [94,95]. Decreased presence of bacteria of the *Lactobacillus* genus (<90%) in the endometrium of women undergoing assisted reproductive techniques (ART) has been associated with recurrent implant failure, reduced pregnancy rates/ongoing pregnancy rates and increased miscarriage rates. In particular, the presence of lactobacilli that produce H_2_O_2_ increases the chance of birth, while the presence of bacteria of the genus *Streptococcus* reduces it [87,88]. In contrast, microbial culture of vaginal and endometrial specimens by women undergoing IVF/ICSI (Intra-Cytoplasmic Sperm Injection) showed that the presence of germs did not benefit a successful pregnancy [96,97].

The follicular fluid has shown the presence of microorganisms, although it is often considered possible to be influenced mainly by the vaginal microbiota. The follicular microbiota possibly affects the reproductive system and gametogenesis. It has been reported that certain bacteria can affect the growth of follicles, even inhibiting their response to gonadotropins. The effect of the microbiota is influenced by the etiology of female infertility [5,98,99]. Finally, ovarian fluid culture from ovulatory follicles of women undergoing IVF has shown that the presence of some bacteria is able to predict the ability of the fetus to implant, as well as to differentiate women according to the cause of infertility, for example polycystic ovaries and endometriosis [100,101]. In fact, *Lactobacillus* spp. has been shown to benefit the effects of assisted reproduction and the women with colonized follicular fluid show changes in the immune response and cytokine profile. The authors therefore proposed the identification of microorganisms in the follicle as a diagnostic test of the true cause of infertility and the prediction of a successful assisted reproduction cycle [102,103]. The bacteria of the genera *Pseudomonas*, *Acinetobacter*, *Vagococcus* and *Sphingobium* in the fallopian tubes and in the follicles is a poor prognostic factor for IVF, and their presence increases the likelihood of bacterial vaginosis in women with tubal infertility [104].

Sperm fluid microbiota is considered to play an important role in male fertility, presence or absence of symptoms of acute or chronic inflammation [60]. It interacts with the microbiota of the female reproductive system and ultimately affects the ability to conceive and birth rates. Sperm microbiota shows lower bacterial colonization compared to vaginal microbiota but has greater diversity [59,105]. Several post-genomic identification studies have shown the presence of bacteria of the genera *Corynebacterium*, *Gardnerella*, *Lactobacillus*, *Prevotella*, *Pseudomonas*, *Streptococcus* and *Veillonella* and from the *Lactobacillaceae* family [106]. Like for the female reproductive system, the presence of *Lactobacillus* species from *Lactobacillaceae* family marks a good quality, and so healthy sperm. The presence of *Pseudomonas* and *Prevotella* shows pathological characteristics that lead to a low quality of sperm [107]. The prostatitis alters the microbiota, reducing the concentration of species from *Lactobacillaceae* family (such as *L. iners*) and that can lead to a low sperm quality. In fact, the effect of prostatitis on sperm is like the effects of bacterial vaginosis on the vaginal microbiota [108,109]. Heterosexual intercourse is the sharing point of the male and female reproductive systems. The microbiota on the skin of the penis and urethra is very similar to the vaginal microbiota of a sexual partner with vaginitis compared to other women with vaginitis [110,111]. During sexual intercourse the sperm are protected from the microbiota of the vagina and specific *Lactobacillus* species which probably support their functionality in the movement during the journey they have to perform [112,113]. Identifying specific groups of bacteria to categorize men as infertile requires further research. Currently no tests are able to determine a threshold for predicting the effect of specific microorganisms on male sperm to be considered infertile. A strong correlation was found between the high concentration of *Anaerocccus* and sperm quality, respectively, suggesting the bacteria from the genera *Lactobacillus*, *Prevotella*, *Haemophilus* and *Pseudomonas* as possible indicators of the investigation of male infertility. The predominant presence of *Pseudomonas* or *Prevotella* species is mainly found in men with poor sperm quality, and the presence of bacteria from the *Lactobacillus* genus prevents the growth of adverse microbes and contributes of good quality sperm [114,115].

### 4.1. Reproductive System Microbiota and Immune Functions during Conception

In both normal conception and ART, the goal is the development of a normal fetus and the birth of a healthy newborn. The immune system of mammals is dependent on and interacts with the human microbiota, suggesting that it undergoes severe changes during pregnancy, which is related to the maternal immune adaptation to the fetus. Major changes are observed in the microbiota of the vagina and intestine [104,116,117]. The immune system during conception and pregnancy detects antigens (PAMPs, DAMPs) mainly via TLR and Nod-like receptors. The semen, the fetus and the placental trophoblast act as antigens. The immune response appears to be activated to initially distinguish the reproductive compatibility as well as the developmental capacity of the fetus [118,119,120,121]. Modification of the maternal microbiota before conception or embryo transfer can therefore play a very important role in the outcome of pregnancy. The microbiota of the vagina affects their ability microorganisms to bypass the cervical mucus barrier and colonize the uterus before pregnancy. Of course, whether this affects peri-implantation processes depends on the microbial composition, genetic background and other factors [121,122]. The uterine microbiota affects the cytokine profile. The fallopian tubes and uterus secrete cytokines and growth factors that affect the fetus and affect its development and adaptation to the uterine environment. Several studies on animals have shown that the Th-2 immune response, and especially the presence of IL-10 and IL-4, is favorable in contrast to the Th-1 response. Also important for blastocyst development is the secretion of growth factors, such as GM-CSF, CSF-1 ane LIF, while TNF-α and IFN-γ inhibit implantation [122,123,124,125,126].

### 4.2. The Role of the Genital Microbiota on Fetus Health

Although the role of the microbiota is studied mainly during implantation and in the early stages of embryonic development, its contribution to the subsequent health of the offspring is important and depends on the method of birth. In assisted reproduction, hormone levels regularly change, but their levels are rarely associated with the microbiota. The drop in the concentration of circulating E2 in the interval between hCG administration and embryo transfer is considered a necessary but not an individual condition to achieve implantation [126,127,128]. A study on ovariectomized rats showed that the concentration of *Lactobacillus* species in the reproductive system depended on estrogen levels. Moreover, the vaginal microbiota, in addition to what it has correlated with estrogen and progesterone levels, appears to affect the outcome of embryo transfer and pregnancy [128,129]. Administration of GnRH for a few months as a therapeutic approach for endometriosis increased vaginal pH significantly in both women with and without endometriosis. A decrease in the levels of beneficial microorganisms and an increase in bacteria of the *Gardnerella* genus and *Escherichia coli* [129,130,131,132]. Clear ART aims not only to conceive and conceive successfully, but also to give birth to healthy offspring. A high percentage of pregnancies from assisted reproduction are multiple, and therefore show increased rates of birth by caesarean section, especially if it has been preceded by precedent caesarean section. Moreover, the probability of performing a cesarean section seems to be higher in infertile women compared to fertile ones, a fact that is also related to the age of the mother [132,133,134]. Newborns begin to install their intestinal microbiota from the first week of birth until one year later. Increased colonization by environmental microorganisms has been associated with birth through caesarean section. An increase in bacteria of the species *Klebsiella*, *Clostridium* and *Enterobacter* has been observed [134,135]. In addition, the cesarean delivery may be responsible for the appearance atopic diseases in childhood. Live birth rate (LBR) has been directly correlated with the presence of species from the *Lactobacillaceae* family in the embryo transfer catheter, and therefore in the reproductive system [91,135,136]. In contrast, the presence of bacterial vaginosis is negatively correlated [134,135,136]. Premature birth also seems to be more related to the bacterial imbalance of the microbiota rather than to the exclusive presence of microorganisms in fetal tissues such as the placenta. Colonization and permanent establishment of urea plasma, and mycoplasma has been associated with premature birth [137,138,139,140].

## 5. The Role of Probiotics as Adjuvants for the Eubiosis in the Urinary Tract

**Figure 4:**
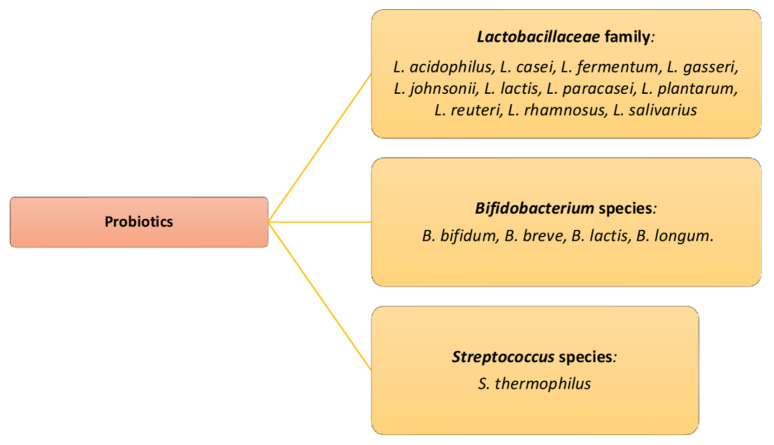
Main commercial probiotic species used.

The microbiota significantly contributes to host resistance to infectious diseases. In addition, changes in the composition of the microbiota are often confused with the disease and in some cases may be the cause of the disease. Probiotic microorganisms are useful to maintain the health balance. The properties of probiotics to intervene in the treatment of various infections in the human organism, therefore also those of the urinary tract have been studied for a long time and are supported by an increasing number of clinical data for specific probiotic strains [22,135,140,141] There is a close correlation, as we have previously noted, between the dysbiosis of the normal microbiota of the urogenital system, which concerns the decrease in the species by *Lactobacillaceae* family, therefore the higher incidence of urinary tract infections. The probiotics microorganism can release antitoxins (serine protease and phosphatase) against toxins of *E. coli* and *Clostridioides difficile*, and they have an immunomodulatory activity (induce two types of cytokines, IL-10 and IL-6) and promote a Th1 immune response. The Lactobacillus spp. are considered beneficial and improve urogenital health through adaptation of the immune system, limiting the growth of pathogenic microorganisms from the rectum and thus involvement in the colonization and survival of beneficial microorganisms. The *Lactobacillus acidophilus* PXN35 and *Lactiplantibacillus plantarum* subsp. *plantarum* (*L. plantarum* PXN47 and *Lacticaseibacillus rhamnosus L. rhamnosus* showed a beneficial antibacterial activity in inhibiting the growth of *E. coli*, inducing the production of mucin (a sticky substance that lines epithelial cells and is known to inhibit adhesion). A randomized, double-blind, placebo-controlled study (RCT) showed that *L. rhamnosus* (1 × 10^9^ CFU/1 billion) and *Limosilactobacillus reuteri Lactobacillus reuteri* (1 × 10^9^ CFU/1 billion), when given orally at specific doses, can restore vaginal populations of *Lactobacillus* spp. after the use of antibiotics or after infections in 96%, compared to 53% of the control groups which prevent urethral infections [140,141,142,143]. A study of 324 females (139 patients with recurrent urinary tract infections and 185 patients without urinary tract infections) showed that a diet with fermented probiotic milk products more than three times per week was associated with a reduced risk of the reinfection relapse. Indeed, probiotic treatment is proved beneficial for many pathologies, and it is suggested that oral doses of approximately 1 × 10^9^ CFU (1 billion) of live bacteria once or twice a week, or more than 1 × 10^8^ CFU (100 million) per day, may be needed to restore and maintain healthy urogenital microbiota microflora (Figure 4) [144,145,146,147,148].

As we mentioned, the vaginal presence of *Lactobacillus* spp. can defend the vaginal mucosa from infections with a coating biofilm. Unfortunately, *Gardnerella*, which is an unwelcome commensal (present in microbiota vaginal type category IV), develops its own biofilm, which, however, is resistant to chemotherapeutic agents such as metronidazole. However, it is vulnerable to the eubiotic biofilm from *Lactobacillus* spp. In this case, the administration of probiotics appears to be more effective than an antibiotic therapy. Long-term use of species from the *Lactobacillaceae* family strains is an alternative to antibiotics. Oral administration of *L. rhamnosus*, *Limosilactobacillus fermentum L. fermentum* and *L. reuteri* appears to increase the endogenous microbiotal strains of *Lactobacillus* spp. [149,150]. In vivo experiments in mice have shown that administration of a *Lactiplantibacillus plantarum* subsp. *plantarum*/*L. plantarum* strain alone or in a therapeutic regimen can inhibit colonization by pathogenic microorganisms. As a result, the microbiota is strengthened and the fertility of mice is restored, as demonstrated by the completion of pregnancy and the birth of newborns [151,152]. The probiotic administration before the start of a cycle of assisted reproduction, but also during it, can improve the rates of implantation, pregnancy and birth. Microbial analysis and administration of probiotic microorganisms can help to restore the vaginal environment and improve the chances of implantation [152,153]. Finally, incubation of sperm with the probiotic strains *Levilactobacillus brevis*/*L. brevis* CD2, *Lactiplantibacillus plantarum* subsp. *Plantarum*/*L. plantarum* FV9, and *Ligilactobacillus salivarius*/*L. salivarius* FV2 results in the maintenance of their viability and motility during in vitro experiments [112,113].

## 6. Conclusions

Current studies now show that the urinary tract is not sterile in healthy people. The microbial community in the urinary tract varies depending on gender, age and disease. The recent advent of new molecular and transgenic techniques has greatly improved our knowledge of the composition of microbial urinary tract communities and also present in various parts of the human body. With this important information, researchers are now ready to translate these findings into a deeper understanding of the complex relationships that exist between the microbiota and its host. These findings further elucidate the role of these microorganisms in human health and their contribution to various diseases, but there many important questions related to the microbial–host relationship remain open.

The microbiota is found both in the lower and upper genitals; it interacts with both the urinary environment, but also with the hormonal environment, and is involved in the fertilized egg implantation, blastocyst formation and the subsequent development of the embryo, as well as in the success of regular and perfect pregnancy of a healthy offspring. The health of the genital system depends on the number of strains of lactic acid bacteria belonging to *Lactobacillus* spp. which maintain eubiosis of the microbiota. In addition, there are clinical studies related to the role of the microbiota in pregnancy, some of which concern women who undergo assisted reproduction techniques (ART). To date, some researchers have tried to modify the genital microbiota to improve reproductive health and the effects of ART and microbiota management can be an important factor in improving reproductive capacity, although the landscape is still cloudy.

It is obvious that we are in the early stage of therapeutic adjuvant approaches, such as prebiotics and probiotics, based on the observation and management of this population or microbiota. The encouraging results of the above research support the possibility that probiotic supplements are beneficial for maintaining the health of the urinary system and certainly give rise to the need for further clinical trials. This opens a broad field of future research to reveal the special interactions between a small and demographically infinite world with the host organism, which will allow us to develop new strategies and therapies for the treatment of various diseases in the context of prevention and design of personalized drugs.. In conclusion, the results of these studies will be the basis for the prevention and design of drugs for the treatment of diseases associated with specific microorganisms of the urinary and genital microbiota together with the individual fertility and successful course of pregnancy.

## Figures and Tables

**Table 1:** The main microbiology laboratory diagnostic tests.

Microbiology Laboratory Diagnostic Tests		
Direct Diagnosis	Rapid Diagnosis	Indirect Diagnosis
Aimed to establish the presence of the pathogen, its identity and its sensitivity to antibiotics, directly in the sample by: microscopic examination * culture examination (isolation) identification (at species level) search for specific antigens (including microbial genome) antibiogram	Useful in severe infections as it allows identification in a short time by: search for antigens search for gene sequences	The objective is to detect the host’s immune response (antibodies) against the infective agent.

* the microscopic examination provides useful information above all for a presumptive diagnosis, which must then be subsequently confirmed.

**Table 2:** The urinary tract microbiota and its homeostatic properties.

Urinary Microbiome and Homeostasis					
It can produce neurotransmitters that interact with the CNS	It can compete with pathogens for common energy sources	It can play a role in the regulation of epithelial molecular biomechanisms and in maintaining the correct structure between its cells	It can produce antimicrobial molecules that kill pathogens	It can strengthen the epithelial defenses and thus the immune system	It can degrade harmful factors (such as some microbial toxins and other).

**Table 3:** The differences in the urinary microbiota between the genders in three studies on urine (samples: * Clean-catch midstream, ** First void).

The Microbiota of the Urinary Tract		
* Healthy women	** Healthy men	* Healthy men and women
*Lactobacillus*, *Prevotella*, *Gardnerella*, *Peptoniphilus*, *Dialister*, *Finegoldia*, *Anaerococcus*, *Allisonella*, *Streptococcus*, *Staphylococcus*	*Lactobacillus*, *Streptococcus*, *Sneathia*, *Mycoplasma, Ureaplasma*	*Jonquetella*, *Parvimonas*, *Proteiniphilum*, *Saccharofermentans* Phyla: Actinobacteria, Bacteroidetes

**Table 4:** The most frequent uropathogens.

Uropathogenic Microorganisms		
Gram (−)	Gram (+)	Fungi
*Escherichia coli*	*Enterococcus* spp.	*Candida* spp.
*Proteus* spp.	*Staphylococcus saprophyticus*	
*Klebsiella* spp.	*Staphylococcus aureus*	
*Citrobacter* spp.	*Streptococcus* spp.	
*Serratia* spp.		
*Acinetobacter* spp.		
*Pseudomonas aeruginosa*		

**Table 5:** Some uncultured bacterial species found in urinary tract.

Sample Source	Species
Voided urine and Transurethral catheter urine sample	Uncultured bacterium BF0002B042, Uncultured *Prevotella* spp., Uncultured bacterium clone HRX_H16

**Table 6:** The five types of vaginal colonization, according to Community State Type (CTS). The *Lactobacillus* spp., which colonize the vagina immediately after birth, creating an acidic environment with the production of a protective for health biofilm.

Frequency of Detected Vaginal Bacteria	
Community State Type (CST)	Dominant Bacteria
1	*Lactobacillus crispatus* 25%
2	*Lactobacillus gasseri* 5%
3	*Lactobacillus iners* 35%
4	Poor in species from *Lactobacillaceae* family, *Gardnerella* 30%
5	*Lactobacillus jensenii* 5%

## Data Availability

All data related to this study are reported in the manuscript.
